# Our New York Letter

**Published:** 1893-03

**Authors:** A. L. Loomis

**Affiliations:** New York


### Correspondence.

### NEW YORK LETTER.

### A CLINICAL LECTURE BY A. L. LOOMIS, M. D., NEW YORK.

GENTLEMEN.—This patient is a man, thirty-four years old, who gives a good family history. His past history is, that he has had no illness other than the ordinary diseases of childhood. His occupation is that of a truck-driver, and his habits those of an excessive drinker. His present illness began three weeks ago. The first thing that he recollected was a shortness of breath on exertion, soon his legs began to swell, particularly after standing up for any length of time. His urine is normal. Notice that his legs pit on pressure.

Now, we must find out the cause of this edema of the legs. It may be the result of a pathological condition of the heart, kidney, or liver. If he had nephritis we would find evidence of it in his urine; as the urine is normal, we exclude the kidney as the cause of the edema; as for the liver, we find nothing, on physical examination, that would warrant the belief that the organ is diseased; besides, he has had no ascites, which he would have had previous to the swelling in the legs if the liver were at fault.

Now, in approaching our diagnosis, we have the following things to consider: first, that the edema pits on pressure, thus proving it to be true edema; second, that the edema disappears when he lies down for any length of time; and third, that it is confined to the legs. We now, naturally, look to the heart, and although the man has complained of no cardiac trouble, he may have had cardiac disease and suffered from it no trouble that would attract his attention.

On examining this patient's pulse, we find it feeble, irregular and intermittent. We also discover a double murmur at the base, but it is slight and does not indicate an extensive lesion. The apex-beat is normal.

Edema of the legs in heart disease is the direct result of a mechanical process. There is, in this case, a stasis of blood in the dependent vessels and a consequent transudation of serum into the adjacent cellular tissue; if it were from kidney disease, it would be due to changes in the blood itself.

The cardiac disease causing this edema is a simple weakness, a feebleness of the muscular power of the heart. We will now consider the nature and cause of this weakness. Alcohol, used in sufficient quantities, acts directly on the nervous system in a deleterious way, and causes alcoholic neuritis; in this disease and in its modifications, the muscles, owing to their depressed nervous supply, suffer from lack of nutrition, hence weaken and atrophy. Now, since alcöhol, used in sufficient quantities, cause this condition, it is reasonable to assume that even in lesser quantities, as has been used by this patient, we get a like condition but to a lesser degree. Of all the muscles of the body, the heart is the most likely and the earliest to succumb to alcohol. The effects of the alcohol upon the nervous system so effect the coronary arteries and aorta that the blood supply to the heart is lessened. First and foremost of the things which lessen the heart's power is alcohol. Its poisonous effects are more pronounced than those of opium or of uremia. Alcoholic degeneration of the heart is a serious condition. When a patient, with such a heart, contracts pneumonia, or any acute disease, his case is grave, because the heart, having been abused and consequently weakened, is called upon to face great odds, and although it may fight vigorously for a time, soon its lessened energies collapse and its beats are stilled.

This then is the condition of the heart in this patient. It is weakened and thus unable to adequately perform its function. As a result there is a stasis of blood in the veins of the legs and a consequent transudation of serum.

We will now consider the treatment of this patient. It would be unwise to treat this as a cardiac disease, pure and simple.

Our first attention must be directed toward the removal of the cause and then to the general nutrition. Alcohol must be stopped. He needs rest and good food in order that his heart may regain its former strength. He should not be permitted to perform any exercise that would overtax his heart, as this would soon produce dilatation, a condition from which he would probably never recover. Keep such a patient in bed, give him tonics and easily digested food, remembering that his heart-trouble is secondary to nervous depression, giving loss of nutrition, and it is this loss of nutrition which we must overcome.

